# Ultrastructural and molecular implications of ecofriendly made silver nanoparticles treatments in pea (*Pisum sativum* L.)

**DOI:** 10.1186/s43141-021-00285-1

**Published:** 2022-01-05

**Authors:** May Labeeb, Abdelfattah Badr, Soliman A. Haroun, Magdy Z. Mattar, Aziza S. El-kholy

**Affiliations:** 1 Botany and Microbiology Department, Faculty of Science, Kafrelsheikh University, Kafr Elsheikh, Egypt; 2 Botany and Microbiology Department, Faculty of Science, Helwan University, Cairo, Egypt; 3 Botany and Microbiology Department, Faculty of Science, Menoufia University, Shebin Elkom, Egypt

**Keywords:** Nanomaterials, Cell structure, Genetic toxicity, Pea plant, ISSR fingerprinting, Isozymes

## Abstract

**Background:**

Silver nanoparticles (AgNPs) are the most widely used nanomaterial in agricultural and environmental applications. In this study, the impact of AgNPs solutions at 20 mg/L, 40 mg/L, 80 mg/L, and 160 mg/L on cell ultrastructure have been examined in pea (*Pisum sativum* L) using a transmission electron microscope (TEM). The effect of AgNPs treatments on the α, β esterase (EST), and peroxidase (POX) enzymes expression as well as gain or loss of inter-simple sequence repeats (ISSRs) markers has been described.

**Results:**

Different structural malformations in the cell wall and mitochondria, as well as plasmolysis and vacuolation were recorded in root cells. Damaged chloroplast and mitochondria were frequently observed in leaves and the osmiophilic plastoglobuli were more observed as AgNPs concentration increased. Starch grains increased by the treatment with 20 mg/L AgNPs. The expressions of α, β EST, and POX were slightly changed but considerable polymorphism in ISSR profiles, using 17 different primers, were scored indicating gain or loss of gene loci as a result of AgNPs treatments. This indicates considerable variations in genomic DNA and point mutations that may be induced by AgNPs as a genotoxic nanomaterial.

**Conclusion:**

AgNPs may be used to induce genetic variation at low concentrations. However, considerations should be given to the uncontrolled use of nanoparticles and calls for evaluating their impact on plant growth and potential genotoxicity are justified.

## Background

In agricultural sector, nanotechnology is a favorable technology for creating huge changes. Nano-based sensors are the ideal approach toward precision farming for monitoring all factors that may improve agricultural productivity. Furthermore, nanotechnology can play a significant role in post-harvest food processing and packaging to reduce food contamination and minimize the waste on delivery and storage [1, 2]. Silver nanoparticles (AgNPs) are the most important nanomaterials and used in about 24% of all nanotechnology applications [3]. Due to their catalytic activity, AgNPs have been used in the manufacturing of insecticides and pesticides [4, 5], the degradation of toxic chemicals [6], and as a disinfectant [7]. The widespread and increasing applications of AgNPs have led to their release into the environment and may easily enter the food chain in several ecosystems and can be transported up to higher-level consumers causing severe lethal effects on non-tolerant species [8–11]. In plants, AgNPs are taken up by plant roots and can be translocated from root to shoot and accumulated in cells, and moved between cells through plasmodesmata [12, 13]. The AgNPs can reach leaves through the flow of water and nutrients by cellular communication and at high concentrations may affect the cellular mechanisms of cell division and repair [14].

It has been reported that AgNPs cause ultrastructural malformation in the cells of different plants such as rice [15], barley [16], *Brassica* [17], tobacco [18–20], and *Scots pine* and oak [21]. Also, [22] reported obvious changes in the anatomy of the lettuce leaves in response to the Ag NPs compared to the control. Patlolla et al. [25] demonstrated that AgNPs reduced mitotic cell division in root tip cells of broad bean and induced chromosomal aberrations and micronuclei production suggesting a disruption in cell cycle and mitosis. Toxicity of AgNPs to cell structure, cell division, and chromosomes has also been reported by [28] in wheat and [29] in pea. Yan and Chen [30] reviewed the underlying mechanism for the impacts of AgNPs phytotoxicity on plants and Badr et al. [31] reviewed the cytogenetics and genotoxic potential of nanoparticles in plants.

The biochemical and molecular responses of plants to AgNPs have been studied in some plants such as rice [32], barley [16], pearl millet [33], tomato [34], and *Psophocarpus tetragonolobus* [35]. Vishwakarma et al. [17] reported that oxidative stress induced by AgNPs leads to DNA degradation and cell death in *Brassica*. Meanwhile, DNA fingerprinting has been used as a biomarker for investigating the genotoxic effect of pollutants on plants [31, 52–54]. ISSR has been used to investigate the mutagenicity of environmental pollutants [39] and as an informative measure of the toxicological impacts on plants exposed to genotoxic material including nanoparticles [40, 41]. The ISSRs markers have been used to indicate genetic stability [35] or genetic variability [42] after exposure to AgNPs.

Pea (*Pisum sativum* L.) is an important legume crop that used as human food, livestock fodder and a source of high protein content, and has a good taste, and digestibility [43, 44]. It is often cultivated for fresh green seeds, dried seeds and, foliage. Pea is the oldest model object of plant genetics and one of the most agriculturally important legumes in the world [45]. This study investigates the effects of AgNPs on the cell ultrastructure of green pea, the expression of α, β esterase, and peroxidase enzymes as well as their impact on genome integrity as measured by ISSR products amplifications and to test the use of ISSR marker polymorphism to detect genetic variation as a result of exposure to AgNPs.

## Methods

### Silver nanoparticles preparation

The silver nanoparticles, used in the current study, were eco-friendly prepared using the gelatine glucose mixture as a reducing/stabilizing agent for silver nitrate and characterized as described in [29].

### Plant material

The study was carried out in the Botany Department, Faculty of Science, Kafrelsheikh University, Egypt. Pea (*Pisum sativum* L. cv Master B) seeds were obtained from the Horticultural Department, Faculty of Agriculture, Kafrelsheikh University, Egypt. Seeds were sterilized using 5% sodium hypochlorite for 10 min and washed several times with distilled water. Healthy seeds were soaked in distilled water as control, and in AgNPs solutions at concentrations of 20, 40, 80, 160 mg/L for 2 h and then germinated on moistened filter papers in sterilized glass Petri-dishes (15 cm) with frequent irrigation by distilled water for the control seedlings or AgNPs solutions (1 ml) every 24 h for 2 weeks at 22 ± 1 °C. Eight seeds were sown in each dish moistened with 5 ml of each treatment with three replicates. Root samples were collected on the 7^th^ day from sowing and leaf samples were collected on the 14^th^ day to study the effect of AgNPs on the cell ultrastructure in root and leaf tissues, respectively. Isozyme analysis was done on the 10^th^ day of germination from roots, while DNA analysis was done using young leaves after 14 days germination. For Fourier-transform infrared spectra (FTIR) analysis and energy-dispersive spectroscopy (EDS), seedlings of 160 mg/L of AgNPs solution were collected, washed with distilled water, separated into roots and shoots, dried in an oven at 80 °C, and well crushed in a mortar to a fine powder.

### FTIR and EDS measurements

FTIR was measured using a JASCO spectrometer (FT/IR-6800) to investigate the functional groups of AgNPs and determine possible binding sites with AgNPs in the root and shoot of pea seedlings. Data were analyzed using the software Origin Professional Program version 8.0. On the other hand, the Energy dispersive spectroscopy was determined using scanning electron microscope (JEOL, SEM - IT100) instrument equipped with EDS at 30 kV to examine the translocation of AgNPs in the root and shoot of seedlings.

### Transmission electron microscope samples preparation

The effect of AgNPs on root and leaf cells ultrastructure was studied by TEM ultrathin sectioning. The samples were prepared using a modified Karnovsky solution [46]. Post-contrast of sample sections were carried out according to [47]. Sections were investigated using TEM JEOL JEM-2100 at 160 kV at the EM Unit, Mansoura University, Egypt.

### Protein extraction for α, β esterase, and peroxidase expression

For these measurements, 200 mg roots of 10-day-old seedlings were powdered and suspended in 500 μl of 0.025 sodium phosphate buffer (pH = 7.25) with 20% (w/v) sucrose, with stirring every 15 min. Samples were centrifuged at 16,000 rpm for 20 min at 4 °C and supernatants were kept at – 20 °C until use. Isozymes were separated using 10% (w/v) polyacrylamide gel according to a protocol proposed by [48], three isozymes were studied (α and β esterase and peroxidase) and detected by a specific stain for each enzyme.

### Detection of α, β esterase enzymes expression

Gels were incubated in 100 ml of 0.05 M phosphate buffer pH = 6 and 0.15 g fast blue B salt was added. α-Naphthyl acetate (0.02 g in 1 ml acetone) was added to the mixture for α-esterase enzyme. While in the case of β-esterase enzyme, β-naphthyl acetate (0.02 g in 1 ml acetone) was added . Gels were incubated in the stain at 37 °C in the dark until bands appeared, then gels were washed with distilled water and fixed in 3% acetic acid to reduce nonspecific background [49].

### Peroxidase enzyme detection

The Gel was incubated in 100 ml of 0.05 M of acetate buffer pH = 5 containing benzidine (0.065 g dissolved in 1ml ethanol), 2 ml of 0.1 M of CaCl_2_ as a coenzyme, and 2 ml of H_2_O_2_ [49]. The gel was incubated at 4 °C in the dark until brown bands appeared, washed by distilled water, and fixed in 50 % glycerol.

### DNA extraction and ISSR fingerprinting

DNA was extracted and purified from leaves of 14 days pea seedlings using the protocol of [50]. 200 mg of fresh young leaves were grinded in 500 μl of CTAB buffer, incubated for 30 min at 55–65 °C, and centrifuged at 10,000 rpm for 8 min. The supernatant was transferred to a clean 1.5 μl Eppendorf tube, half volume of chloroform-isoamyl (24:1) was added, centrifuged, and the supernatant was transferred to a clean 1.5 μl Eppendorf tube. An equal volume of cold isopropanol was added to precipitate DNA, samples were inverted very slowly, and then were centrifuged. The supernatant was removed and the precipitate was washed by cold 70% ethanol, centrifuged, and DNA was re-precipitated by adding 750 μl cold absolute ethanol and 100 μl 3 M sodium acetate; precipitate was washed by cold 70% ethanol and centrifuged. The supernatant was removed, DNA was left to air dried, resuspended in deionized water, and stored at − 20 °C. DNA quantified using a nano-drop ND-100 P330 spectrophotometer (IMPLN) Germany, and visualized on 1% agarose gel.

For ISSR fingerprinting, 22 primers (Table 1) were used in 20 μl reaction volume containing 1 μl from the primer, 2 μl genomic DNA (20 ng), 10 μl Dream Taq Green PCR Master MIX (Thermo Fisher Scientific, Inc.) consisting of (Dream Taq DNA polymerase, 2x buffer and 4 mM MgCl_2_) and 7 μl dd.H_2_O. Polymerase chain reaction (PCR) was performed as follows: initial denaturation at 95 °C for 5 min, 40 cycles of denaturation at 95 °C for 1 min, annealing at 45 °C for 40 s, extension at 72 °C for 1 min, and a final extension at 72 °C for 5 min using a Primus 25 advanced® cycler machine. DNA was visualized using 10 μl from PCR products on 1.6% agarose in TBE buffer with ethidium bromide at 100 V for 1 h and photographed by the Gel Documentation system (WiseDoc®, WGD-30, DATHAN Scientific, Co., Ltd.).

**Table 1:** ISSR primers, sequences, and percentage of polymorphism for *Pisum sativum* treated with distilled water as control, 20, 40, 80, and 160 mg/L of AgNPs solutions

No.	ISSR primers	Sequence (5′-3′)	Total bands	Range of size (bp)	Monomorphic bands	Polymorphic bands	% Polymorphism
1	UBC 810	(GA)_8_T	5	354–925	3	2	40
2	UBC 811	(GA)_8_C	3	561–948	1	2	66.67
3	UBC 812	(GA)_8_A	4	877–1427	3	1	25
4	UBC 825	(AC)_8_T	5	319–551	4	1	20
5	UBC 834	(AG)_8_YT	8	194–800	4	4	50
6	UBC 835	(AG)_8_YC	5	295–688	4	1	20
7	UBC 836	(AG)_8_YA	6	253–1362	3	3	50
8	UBC 840	(GA)_8_YT	7	232–737	3	4	57.14
9	UBC 841	(GA)_8_YC	7	262–987	6	1	14.29
10	UBC 842	(GA)_8_YG	4	248–710	2	2	50
11	UBC 844	(CT)_8_RC	4	286–867	2	2	50
12	UBC 845	(CT)_8_RG	9	199–968	5	4	44.44
13	UBC 847	(CA)_8_GC	3	437–700	2	1	33.33
14	UBC 855	(AC)_8_YT	8	385–1398	6	2	25
15	UBC 856	(AC)_8_YA	–	–	–	–	–
16	UBC 857	(AC)_8_YG	–	–	–	–	–
17	UBC 873	(GACA)_4_	5	354–696	2	3	60
18	UBC889	DBD(AC)7	–	–	–	–	–
29	UBC 898	(CA)_6_RY	–	–	–	–	–
20	UBC 899	(CA)_6_RG	–	–	–	–	–
21	844 A	(CT)_8_AC	4	274–815	2	2	50
22	HB 11	(GT)_6_CC	8	294–1376	4	4	50
Total			95		56	39	41.52

### Data analysis

The molecular sizes of DNA ISSR markers expressed as bands on the agarose gel were determined by Lab-image program version 7.1.3 [51]. Bands were scored in binary matrices as 1 for presence and 0 for absence and similarity between plants exposed to different concentrations of AgNPs were estimated using Dice coefficient of similarity [52] using the NTSYS-pc software version 2.02 [53]. Construction of a distance tree illustrating the distance among the studied plants was performed using the unweighted pair group method using the arithmetic average (UPGMA) [54] as implemented in the NT-SYS-pc.

## Results

### FTIR analysis and EDS

**Fig. 1:**
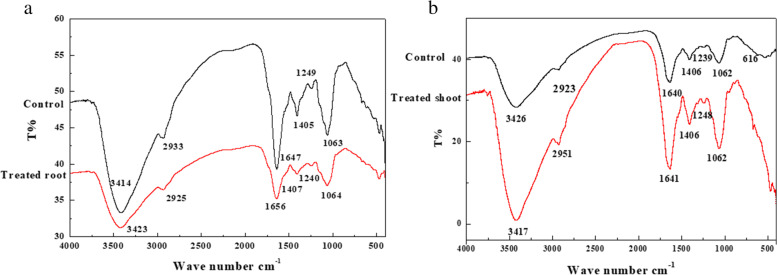
FTIR spectra in seedlings of pea. **a** Roots. **b** Shoots indicating comparison between control seedlings and seedlings germinated in 160 mg/L of AgNPs solution

**Fig. 2:**
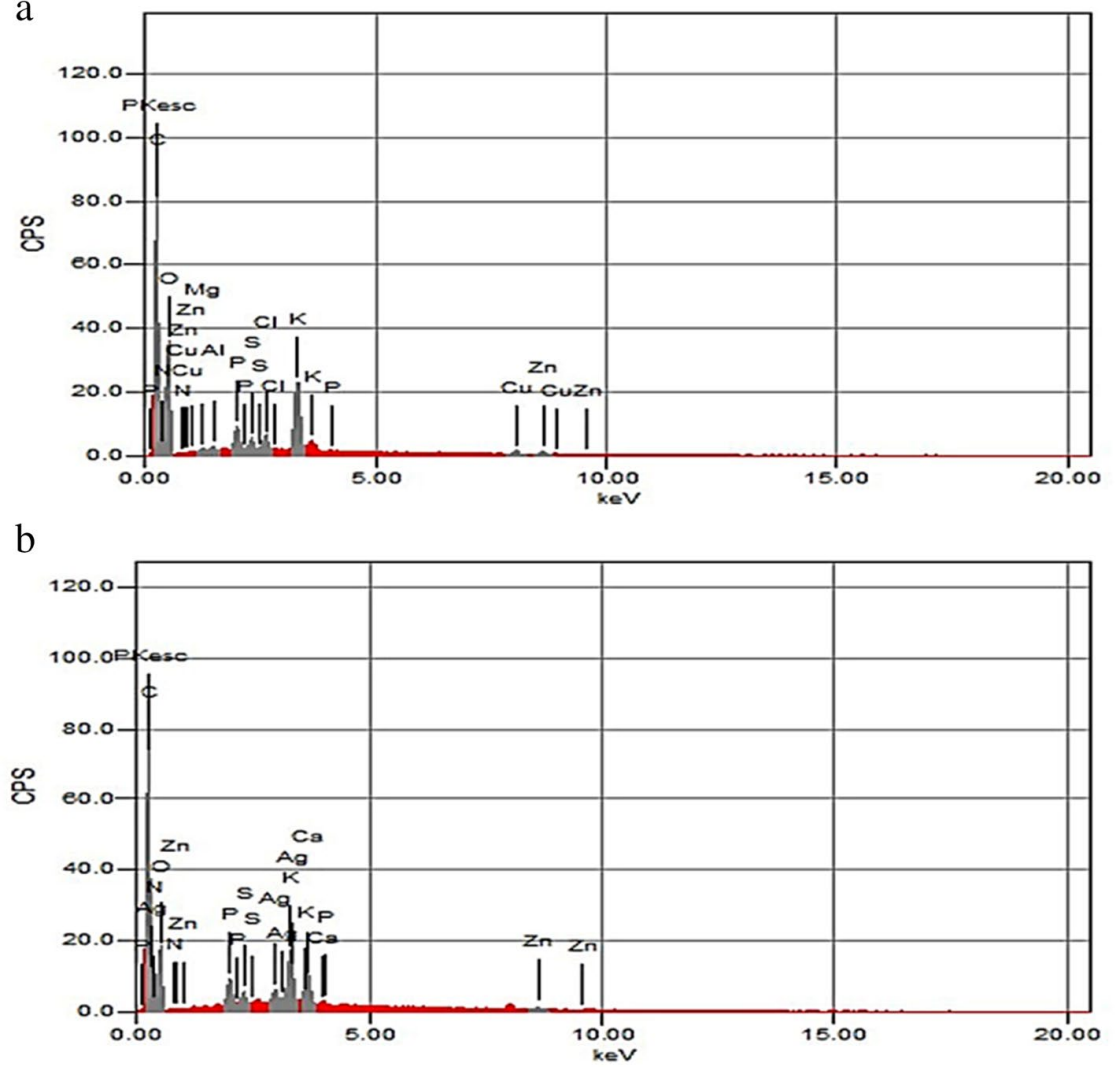
SEM-EDS of roots in pea seedlings. **a** Control roots. **b** Roots treated with 160 mg/L of AgNPs solution

**Fig. 3:**
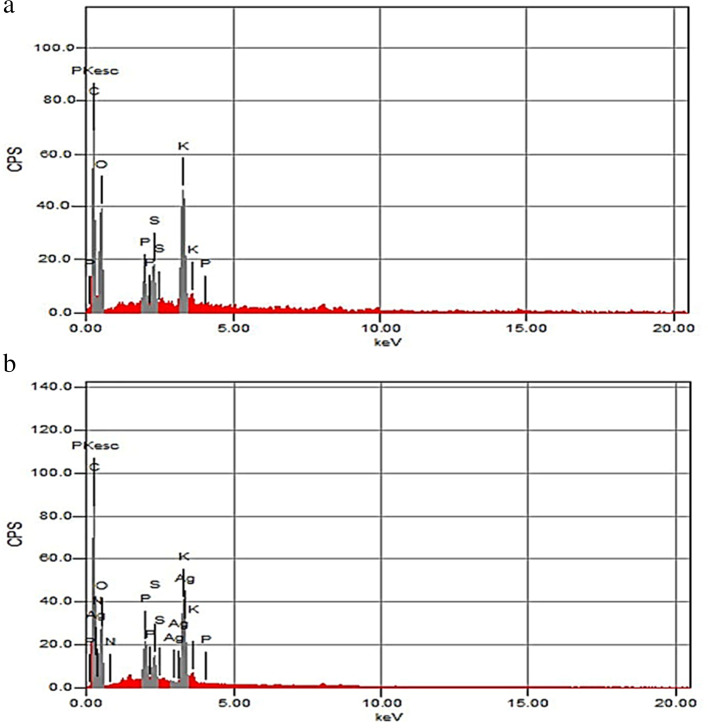
SEM-EDS of shoots in pea seedlings. **a** Control shoots. **b** Shoots treated with 160 mg/L of AgNPs solution

The possible biomolecules which interacted with nanoparticles inside the treated and untreated root and shoot cells were identified by FTIR spectra. The FTIR spectrum of roots in control seedlings located in the region of 500–4000 cm^−1^ showed peaks of 3414, 2933, 1647, 1405, 1249, and 1063 cm^−1^ (Fig. 1a), while treated roots showed peaks of 3423, 2925, 1656,1407, 1240, and 1064 cm^−1^. The FTIR spectrum of shoots in control located in the region of 500–4000 cm^−1^ showed peaks of 3426, 2923,1640, 1406, 1239, and 1062 cm^−1^ (Fig. 1b), while shoots of treated seedlings showed peaks of 3417, 2951, 1641, 1406, 1248, and 1062 cm^−1^. The major molecules are carbonyl, hydroxyl, amino, and carboxyl. The peaks at 3414, 3417, 3423, and 3426 cm^−1^ characterize N-H and O-H groups and the peaks at 2923, 2925, 2933, and 2951 cm^−1^ are attributed to C-H stretching vibrations of –CH_3_ and –CH_2_ groups. The peak at 1640 cm^−1^ can be assigned as a peak of carbonyl groups but the peaks at 1062, 1063, and 1064 cm^−1^ are due to C-O group or polysaccharides. The peak at 1641 cm^−1^ can be assigned as a peak of the C=O group and the peaks at 1405, 1406, and 1407 cm^−1^ can be attributed to amino-substituted alkyl group. The peaks at 1647 and 1656 cm^−1^ are due to C=O, C=N, and, C=C groups. The peaks at 1239, 1240, 1248, and 1249 cm^−1^ are related to C–OH group, C–H stretching vibrations, N–H bending, and −CH3 wagging. The SEM-EDS analysis confirmed the presence of elemental silver signal of the AgNPs inside both root and shoot (Figs. 2 and 3) respectively in plants treated with 160 mg/L AgNPs compared to control.

### TEM images

**Fig. 4:**
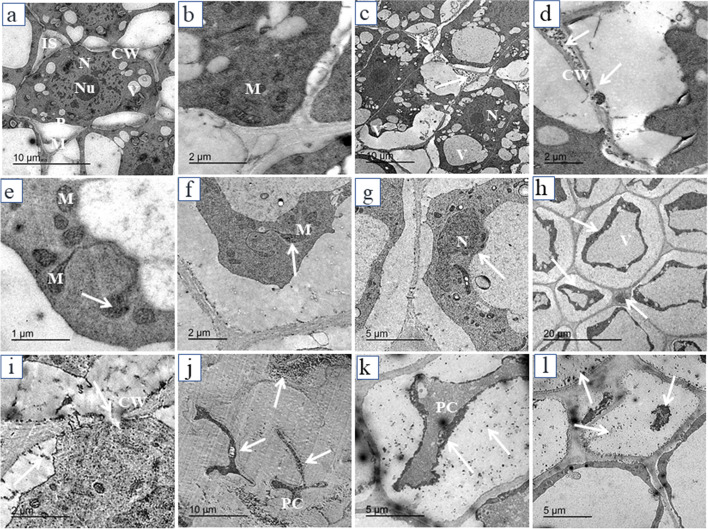
Electron micrographs of transverse sections of the apical meristematic zone of pea root: **a**, **b** control; **c** 20 mg/L; **d**–**h** 40 mg/L; **i**, **j** 80 mg/L; **k**, **l** 160 mg/L AgNPs. CW-cell wall; PM-plasma membrane; V-vacuole; N-nucleus; NU-nucleolus; IS-intercellular spaces; PC-plasmolyzed cell; M-mitochondrion. White arrows refer to affected cell structures and black arrows refer to electron-dense particles

TEM micrographs of transverse sections of the apical meristematic zone of pea roots of control seedlings and seedlings exposed to AgNPs treatments are illustrated in Fig. 4. One of the major impacts of AgNPs treatments is the depositions of small dense particles less than 15 nm on the cell wall and plasmodesmata and also the aggregation of these particles in the intercellular spaces between cells. The presence of these particles caused plasmolysis, vacuolization inside the cell, and breakage in the cell wall. White arrows refer to affected cell structures and black arrows refer to electron-dense particles. Control root cells are showed by Fig. 4a, b and intercellular spaces filled with electron-dense particles (black arrow) and vacuolizations are illustrated in Fig. 4c (20 mg/L). Breakage in the cell wall is shown in Fig. 4d, i (white arrows) as induced by 40 mg/L and 80 mg/L AgNPs, respectively. The number of mitochondria malformation increased by increasing the concentration of AgNPs, the shape of mitochondria was changed from circle or oval in control root cells (Fig. 4b) to pleomorphic shape in treated roots with 40 mg/L AgNPs (Fig. 4e, f). Malformation of the nucleus and cell plasmolysis were also commonly induced by 40 mg/L (Fig. 4g, h). Highly plasmolyzed cells with electron-dense particles by 80 mg/L and 160 mg/L AgNPs are illustrated in Fig. 4j, k respectively. Degradation of cells was shown in Fig. 4l by 160 mg/L. In general, the incidence of cellular structural damage increased by increasing the concentration of AgNPs.

**Fig. 5:**
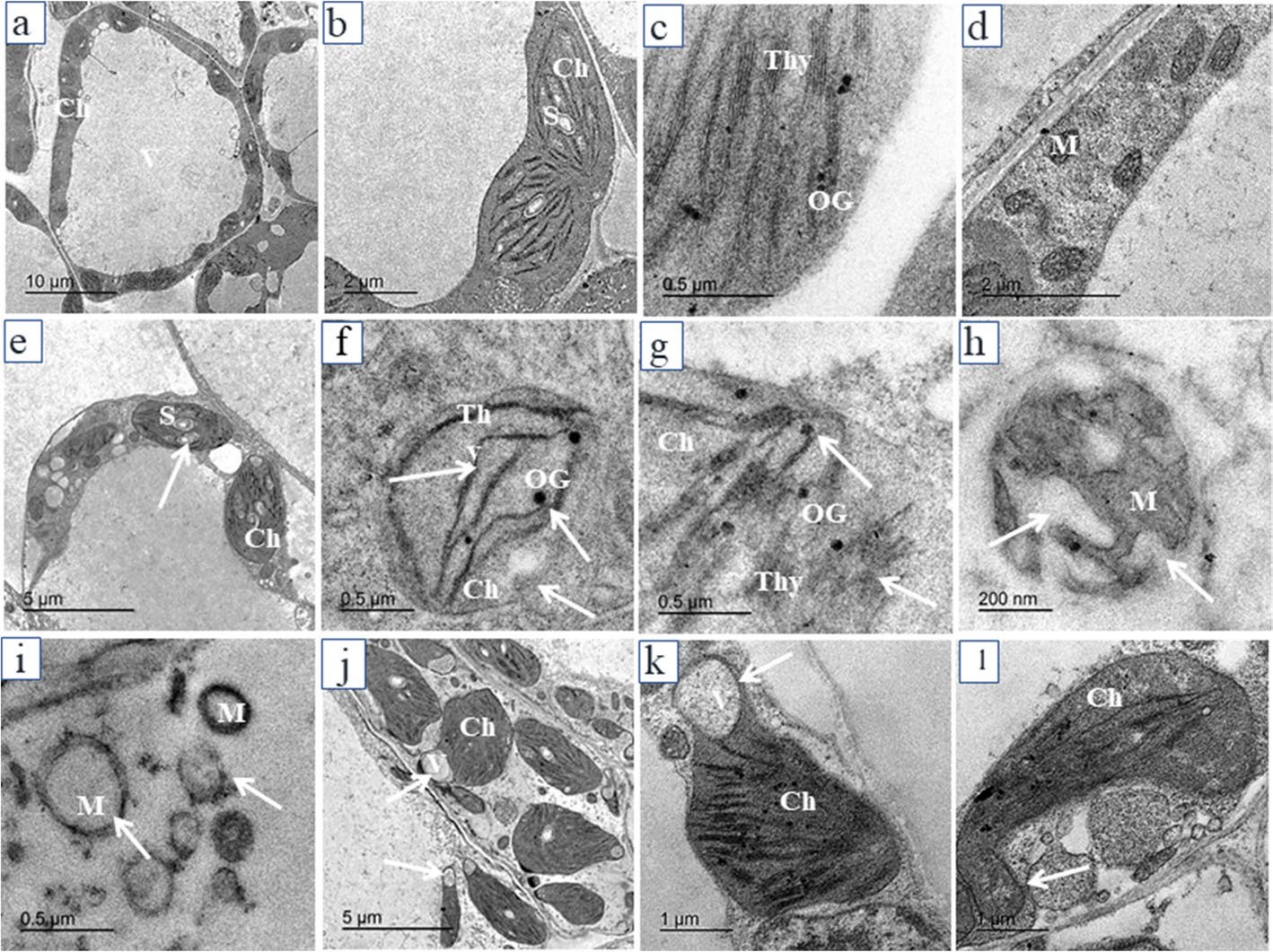
Electron micrographs of transverse sections of leaves of pea plants: **a**–**d** control; **e** 20 mg/L; f**–h** 40 mg/L; **i**, **j** 80 mg/L; **k**, **l** 160 mg/L AgNPs. M-mitochondrion; Ch-chloroplast; S-starch grain; OG-osmiophilic globule (plastoglobuli), Thy-thylakoids. White arrows refer to affected cell structures

TEM micrographs of transverse sections of control leaves of seedlings germinated and grown in distilled water illustrated in Fig. 5a–d. The TEM leaf cells from plants subjected to AgNPs treatments are illustrated in Fig. 5e–l. All AgNPs treatments affected cell shape and the structure of cell organelles especially chloroplast and mitochondrion. The number of chloroplasts also decreased gradually by increasing the concentration of AgNPs. The increase of the starch grains number by 20 mg/L AgNPs is shown in Fig. 5e (white arrow). Destruction in the chloroplast membrane associated with reduced grana lamella and disturbance of thylakoids and increasing number and size of plastoglobuli induced by 40 mg/L AgNPs are shown in Fig. 5f, g. Damage of mitochondrial membrane and reduction in the number of cristae (Fig. 5h) were induced by 40 mg/L. Disappearance of mitochondrial cristae, the irregular shape of cells and chloroplasts with vacuoles and irregular stacking of chloroplasts were sometimes observed by 80 AgNPs (Fig. 5i, j). Malformations in chloroplast shape that appeared with tail and disturbance of thylakoids are shown in Fig. 5k, l (160 mg/L AgNPs).

### Isoenzymes analysis

**Fig. 6:**
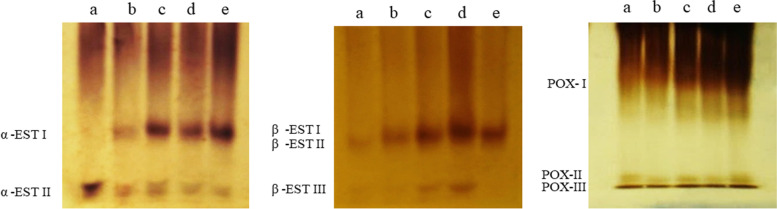
Expression of α-estrase (α-EST), β-estrase (β-EST), and peroxidase (POX) zymogrames in protein of treated pea roots: **a** control, **b** 20 mg/L AgNPs, **c** 40 mg/L AgNPs, **d** 80 mg/L AgNPs, **e** 160 mg/L AgNPs

Variations in the patterns of α-EST I and EST II expression, as isoforms, in treated samples compared to the control are illustrated in Fig. 6. The α-esterase enzyme was expressed as two isoforms in treated samples compared to the control which was expressed as one isoform (α-EST II). The α-EST I isoform was induced by all treatments and its intensity increased by increasing the concentration of AgNPs and was absent in the control. The isoform (α-EST II) has a high intensity in control and becomes faint in samples exposed to AgNPs, its intensity decreased by increasing AgNPs concentration. The β-esterase enzyme was represented by three isoforms; β-EST I was expressed in all treated samples but was absent in the control, β-EST II isoform appeared only in control and is not expressed in the treated samples, while β-EST III isoform appeared very faint in control and 20 mg/L, faint in 40 mg/L, more intensity and thickness in treatment 80 mg/L, and absent in 160 mg/L of AgNPs. Peroxidase enzyme was expressed as three isoforms that appeared in all samples but varied in intensity, the POX I appeared the most intense.

### Impact of AgNPs on ISSR fingerprinting

**Fig. 7:**
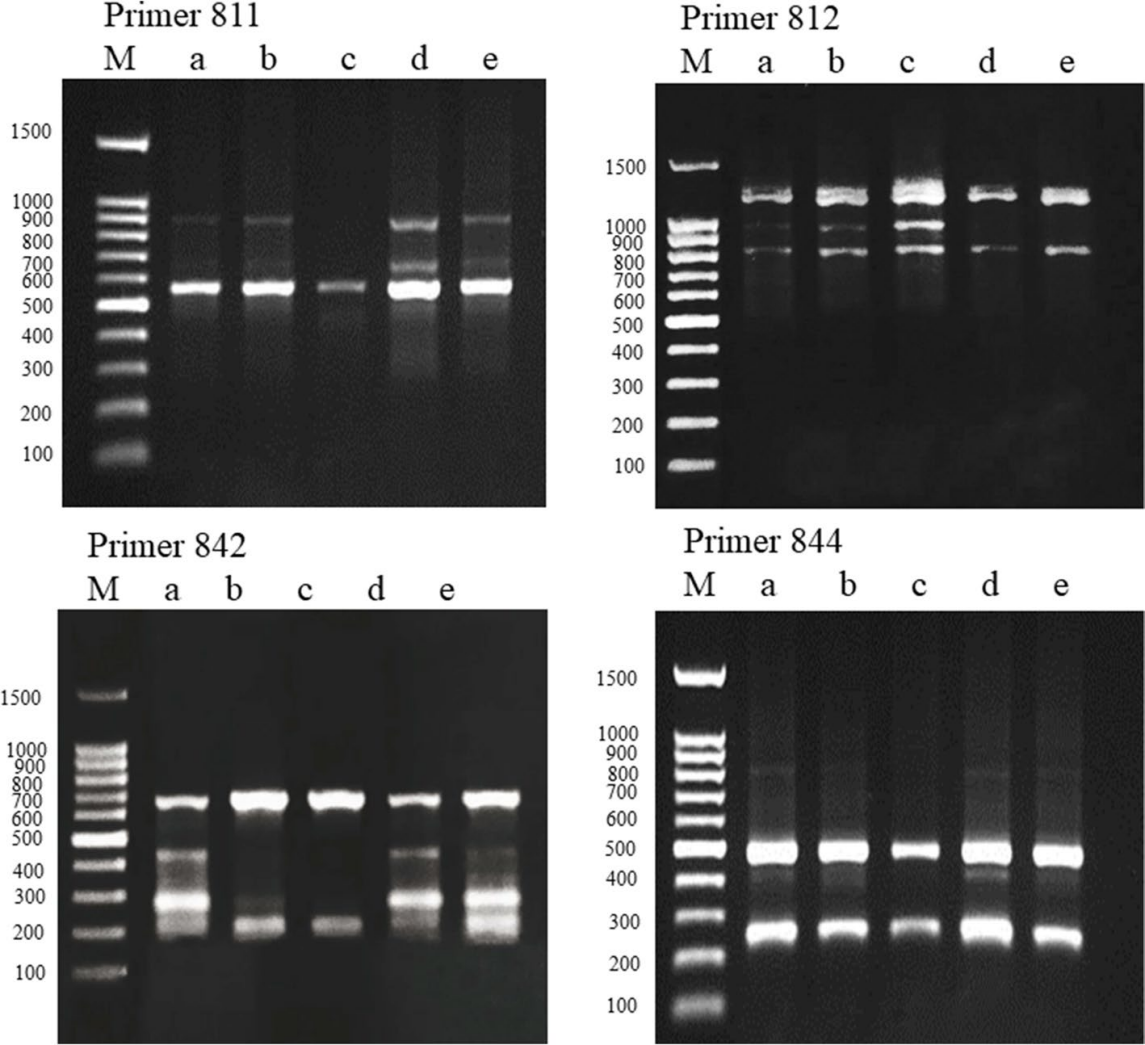
ISSR profile of **a** control pea plants and plants exposed to **b** 20 mg/L AgNPs, **c** 40 mg/L AgNPs, **d** 80 mg/L AgNPs, and **e** 160 mg/L AgNPs using 4 primers, M = standard DNA molecular size marker

**Fig. 8:**
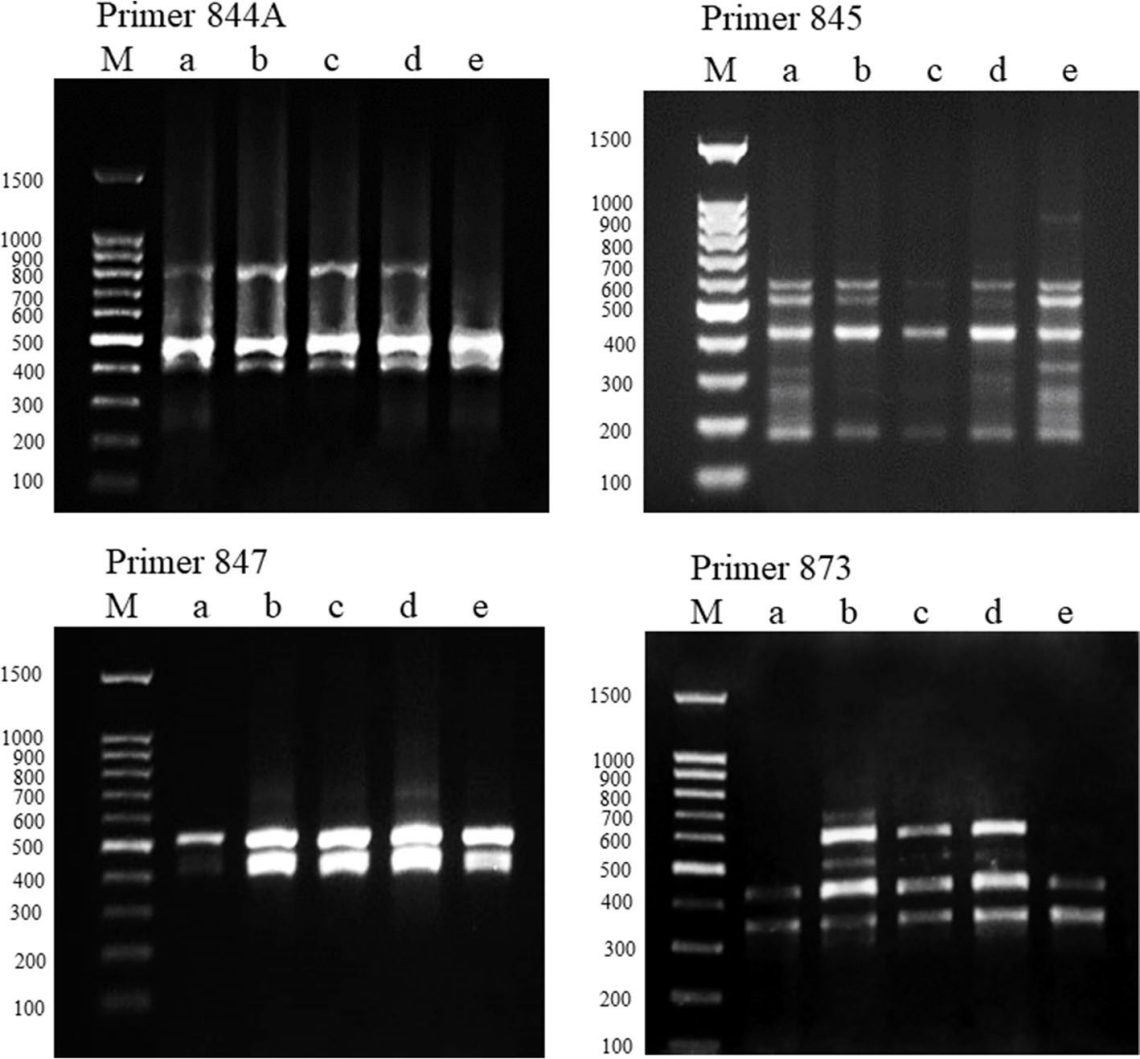
ISSR profile of **a** control pea plants and plants exposed to **b** 20 mg/L AgNPs, **c** 40 mg/L AgNPs, **d** 80 mg/L AgNPs, and **e** 160 mg/L AgNPs using 4 primers, M = standard DNA molecular size marker

Seventeen primers out of tested 22 produced a total of 95 bands and the percentage of polymorphism for all primers was 41.52% (Table 1). The highest percentage of polymorphism (66.67%) was recorded by the primer UBC 811. The ISSR eight finger-printing profiles representing examples of primers tested for control and treated pea seeds are illustrated in Figs. 7 and 8. The used ISSR primers showed that new bands were induced in the treated samples that were absent in the control such as bands formed by primer UBC 847 with size (700 bp) and primer UBC 873 with size (520,613, and 696 bp). On the other hand, bands present in the control are absent in the treated samples as produced by primers UBC 842 with size (293 and 452 bp) and 844A with size (274 bp). Markers produced by some primers such as UBC 812 with size (1068 bp) and UBC 873 with size (520, 613, and 696 bp) were absent in samples treated with high concentrations of AgNPs solutions. Other bands that were absent in samples exposed to low concentrations of AgNPs were recorded at high concentrations as primers UBC 811 with size (692 and 948 bp), UBC 842 with size (293 and 452 bp), and UBC 845 with size (220, 311, and 323 bp).

**Fig. 9:**
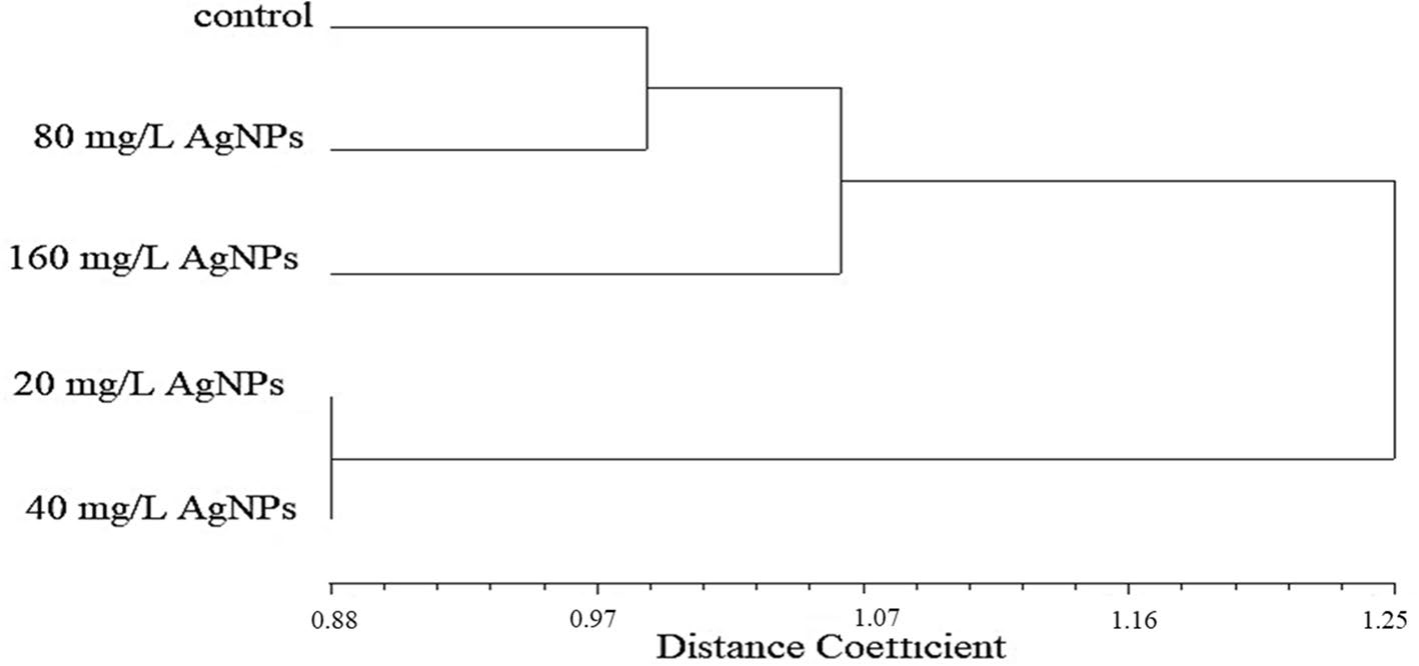
Cluster dendrogram showing the differentiation among control pea plants and plants treated with 20 mg/L AgNPs, 40 mg/L AgNPs, 80 mg/L AgNPs, and 160 mg/L AgNPs based on ISSR fingerprinting

The effects of AgNPs concentrations on the appearance (a) and disappearance (b) of ISSR bands in pea plants treated with 20 mg/L, 40 mg/L, 80 mg/L, and 160 mg/L AgNPs compared to the control are illustrated in Table 2. The treatment with 20 mg/L AgNPs recorded the highest number of appearance of new bands (11 bands), while 40 mg/L recorded the highest number of disappearance of bands (17). Both concentrations resulted in more variations in the ISSR profiles than the 80 mg/L and 160 mg/L. A cluster analysis constructed using the UPGMA algorithm also clearly shows the distinction of plants exposed to the 20 mg/L and 40 mg/L AgNPs solutions from the control plants and plants exposed to the 80 mg/L and 160 mg/L concentrations of the AgNPs solutions (Fig. 9).

**Table 2:** The effects of different AgNPs concentrations on the appearance (a) and disappearance (b) of ISSR bands in treated pea plants

No.	ISSR primers	Control	20 mg/L AgNPs		40 mg/L AgNPs		80 mg/L AgNPs		160 mg/L AgNPs	
			a	b	a	b	a	b	a	b
1	UBC 810	3	1	0	0	0	2	0	2	0
2	UBC 811	3	1	0	0	2	2	0	2	0
3	UBC 812	4	0	0	0	0	0	1	0	1
4	UBC 825	4	0	0	0	0	1	0	1	0
5	UBC 834	5	3	0	3	0	0	1	2	0
6	UBC 835	4	0	0	0	0	1	0	1	0
7	UBC 836	4	1	1	2	1	0	1	0	1
8	UBC 840	7	0	4	0	4	0	1	0	0
9	UBC 841	6	0	0	0	0	1	0	0	0
10	UBC 842	4	0	1	0	2	0	0	0	0
11	UBC 844	4	0	0	0	2	0	0	0	0
12	844 A	4	0	1	0	1	0	0	0	0
13	UBC 845	7	0	2	0	2	0	1	1	1
14	UBC 847	2	1	0	1	0	1	0	0	0
15	UBC 855	8	0	1	0	0	0	0	0	2
16	UBC 873	2	3	0	2	0	2	0	0	0
17	HB 11	7	1	0	0	3	0	0	0	0
Total			11	10	8	17	10	5	9	5
Total a+b			21		25		15		14	
Total bands		78	79		69		81		80

## Discussion

Data of FTIR of root and shoot of 14-day-old seedlings showed slight shifts in positions and variations in peaks intensity between the control and treated plants. The peaks at 3414, 3417, 3423, and 3426 cm^−1^ can be attributed to N-H and O-H stretching vibrations and the peaks at 2923, 2925, 2933, and 2951 cm^−1^ are related to C-H stretching vibrations of –CH_3_ and –CH_2_ functional groups. On the other hand, the peak at 1640 cm^−1^ is due to carbonyl groups stretching from aldehydes and ketones whereas the peaks at 1062, 1063, and 1064 cm^−1^ can be assigned as peaks of C-O functional group and may be considered as characteristic peaks for polysaccharides [55]. The peak at 1641 cm^−1^ is due to the C=O group and the peaks at 1405, 1406, and 1407 cm^−1^ are due to amino-substituted alkyl group [56]. The peaks at 1647 and 1656 cm^−1^ can be attributed to C=O, C=N, and C=C groups [57]. The peaks at 1239, 1240, 1248, and 1249 cm^−1^ are assigned to C–OH stretching vibrations, C–H stretching vibrations, N–H bending, and −CH3 wagging, respectively [58]. This variation in intensity and shifts in positions of peaks confirms that functional groups like amine, hydroxyl, carboxyl, carbonyl, and others are involved in binding of nanoparticles to the cells [15]. The possible mechanisms of nanoparticles adsorption may be due to surface precipitations, complexation with functional groups, physical adsorption, ionic exchange, and chemical reaction with surface sites [59].

EDS analysis confirmed the presence of elemental silver signal of the silver nanoparticles inside both root and shoot of pea. The AgNPs are able to enter cells through endocytosis and react with different cell components and bind to biomolecules through their sulfhydryl groups [60]. In the current study, depositions of AgNPs on the cell wall, plasmodesmata, and their aggregation in the intercellular spaces were observed following exposure to different AgNPs treatments in both root and shoot cells. Castro-González et al. [14] documented that AgNPs were observed in epidermis cells of stevia stem in vitro, within vascular bundles and in intermembrane spaces and in ribs and stomata of leaves by fluorescence microscopy. Geisler-Lee et al. [13] indicated that AgNPs enter root tips at an early stage after exposure and gradually moves in vascular tissue and throughout the whole plant from root to shoot. The AgNPs might take apoplastic pathway and can be translocated from root to endodermis or vascular bands through extracellular spaces of the cells with the xylem which is considered an important vehicle in the distribution and translocation of minerals in plants [61]. The AgNPs are accumulated on the plasmodesmata indicating that silver nanoparticles enter the cell through plasmodesmata [62]. The aggregation of AgNPs on cell walls and plasmodesmata may block the intercellular spaces and subsequently reduce or inhibit nutrients translocation to cells [13]. Mehrian and De Lima [63] suggested three mechanisms for the entry of nanoparticles into the cells. Firstly, nanoparticles move through the cell membrane by direct diffusion; secondly, through endocytosis and thirdly, enter through channels using membrane transporter proteins. The AgNPs may be accumulated in cell vacuoles leading to vacuolization in root cells [18]. Malformation of the nuclear shape may be due to invaginations caused by incomplete endocytosis of AgNPs through the nuclear envelope [29].

In the current study, root growth was more affected by AgNPs than shoot growth and root cells were highly plasmolyzed and degraded by higher concentrations of AgNPs. Hasan et al. [22] found the same results where AgNPs caused cell death at 100 ppm of the concentration on the lettuce. The results also showed an increasing number of starch grains in the chloroplasts at the low concentration of AgNPs (20 mg/L) and decreased at higher concentrations. These results are consistent with the results of [64] in *Vicia faba* seedlings after seed priming by 10 ppm of AgNPs. Increasing starch grains in leaf cells may be the result of increasing photosynthetic machinery and can stimulate the accumulation of starch as a defense response against AgNPs stress [21]. However, number, shape, and size of chloroplasts were changed and the presence of spaces between thylakoids in chloroplasts was recorded at high concentrations indicating the toxic effects of AgNPs on leaf cells. Changes in chloroplast size and shape and chloroplast rupture was observed in tobacco seedlings [18, 19]. The irregular shape of chloroplasts with protrusions and less stacking of grana was also shown in broad bean seedlings after seed priming with high concentrations of AgNPs [64]. Plastoglobuli increased in number and size in leaf cells and [20] found the same results where leaves of tobacco plants treated with AgNP-PVP and AgNP-CTAB were containing thinner and longer chloroplasts and large plastoglobules compared to the control. These structures may be associated with senescing of chloroplasts [65] play a role in the breakdown of carotenoids and oxidative stress defense [66]. Meanwhile, the destruction of the mitochondrial structure is a physiological dysfunction [16] which may affect the respiration chain resulting in reactive oxygen species production (ROS). Mehrian and De Lima [63] assumed that nanoparticles resulted in the formation of H_2_O_2_ reactive oxygen species which reacts with AgNPs to form silver ion (Ag^+^) which causes disruption of mitochondria and other cell components and their functions. Grzelaka et al. [67] reported that damaging of mitochondria and leakage of ROS is the mechanism for the toxicity of AgNPs.

The activity of the enzymes α, β esterase, and peroxidase increased by increasing concentration of AgNPs in agreement with the results of some authors on some plants such as [68] on *Musa acuminate*, [33] on *Pennisetum glaucum*, [34] on tomato. Also, [35] demonstrated that priming seed treating of *Psophocarpus tetragonolobus* plants by AgNPs enhanced antioxidant enzymes (peroxidase, ascorbate peroxidase, superoxide dismutase, and catalase). Increasing the activity of antioxidant enzymes are strategies to tolerate oxidative stress [69]. Mattson [70] reported that toxic metals directly or indirectly trigger the generation of ROS, acting as signals which stimulate the activation of genes during the detoxification of ROS. The increased expressions of isozymes in our study may be indicative of activation of plant defense mechanisms to tolerate stress imposed by AgNPs [32]. Increasing ROS may be the cause of decreasing growth of seedlings [33]. Also, [9] demonstrated that a decline in growth may be due to the destructive effects of ROS on the photosynthetic machinery which may be involved in oxidative stress.

The impact of AgNPs on genome stability in pea seedlings was detected by ISSR markers fingerprinting which showed polymorphism among control and treatments especially following exposure to the treatment of 20 mg/L and 40 mg/L AgNPs. The polymorphism due to the presence or absence of DNA loci between the samples may be the result of DNA damage that may be attributed to point mutations as a result of damage to DNA by AgNPs interaction with the phosphorus of DNA molecule [71]. Gain or loss of loci was detected by using ISSR markers in Chrysanthemum plants treated by 20 mg/L AgNPs [42]. Also, [72] concluded that exposure to AgNPs increases the ISSR polymorphism which could be useful to promote the genetic variability of *Vanilla planifolia*. The changes in the ISSR profiles may be a consequence of the increase in ROS which enhances genomic DNA damages through induction of point mutations thereby leading to ISSR polymorphism. The ISSR marker fingerprinting variation induced by AgNPs profiles demonstrated a consistent increase in polymorphism by the increase in the concentration of AgNPs.

The treatment with 20 mg/L AgNPs resulted in the highest number of loci gain (11 new bands), while the 40 mg/L resulted in the highest number of loci loss (17 absent bands). Both concentrations resulted in more variation in the ISSR profiles than the 80 mg/L and 160 mg/L. 

This is indicated by cluster analysis that clearly showing the distinction of plants exposed to the 20 mg/L AgNPs and 40 mg/L from the control plants and plants exposed to the 80 mg/L and 160 mg/L of the AgNPs solutions. Also, [41] reported a consistent increase in ISSR polymorphic bands in tomatoes by the increase in the concentration of AgNPs. On the other hand, priming seeds of *Psophocarpus tetragonolobus* with AgNPs led to few numbers of new bands, although, in some plants, inflorescence color or shape was altered without major change at the genetic level indicating the possible epigenetic action of AgNPs such as DNA acetylation and/or methylation [35, 73]. Molecular markers contribute to a better understanding of the damage caused by these genotoxins and reveal a promising strategy for prospective studies of the toxic effects of environmental pollutants [74]. For the ISSR marker, the greatest effect observed was band loss by the majority of the applied AgNPs treatments, which could be associated with unrepaired DNA damage hindering the amplification of the sampled sites as well as point mutations at the annealing site [75]. This marker has been used to reveal band loss and gain effects in toxicity studies of a variety of compounds, such as triazoles in *Allium cepa* [76].

## Conclusion

AgNPs induced damage in pea seedlings’ root cells, illustrated by TEM images, as plasmolysis, vacuolization inside the cell, and breakage in the cell wall. Incidences of root mitochondria and nucleus malformation increased by increasing the concentration of AgNPs. In leaf cells, all AgNPs treatments affected cell shape and cell organelles especially chloroplast and mitochondrion. The treatment with 20 mg/L increased the number of starch grains in the chloroplast. The expressions α, β esterase, and POX were slightly changed and considerable polymorphism in ISSR profiles revealed the considerable impact of AgNPs on genome stability and illustrated genotoxic effects of silver nanoparticles on pea seedlings. The 20 mg/L AgNPs resulted in ISSR loci gain while the 40 mg/L resulted in loci loss. In brief, the AgNPs may be used to induce genetic variation at low concentrations that may be used in plant pre-breeding to induce mutations. However, considerations should be given to the uncontrolled use of nanoparticles. We recommend studying the genetic variations in the second generation of pea plants treated with AgNPs.

## Data Availability

Not applicable.

## Abbreviations

AgNPs: Silver nanoparticles
TEM: Transmission electron microscope
EST: Esterase
POX: Peroxidase
ISSRs: Inter-simple sequence repeats
FTIR: Fourier-transform infrared spectra
EDS: Energy-dispersive spectroscopy
SEM: Scanning electron microscope
PCR: Polymerase chain reaction
UPGMA: Unweighted pair group method using the arithmetic average
ROS: Reactive oxygen species
